# Molecular basis of ventricular arrhythmogenicity in a Pgc-1α deficient murine model

**DOI:** 10.1016/j.ymgmr.2021.100753

**Published:** 2021-04-09

**Authors:** Khalil Saadeh, Karan R. Chadda, Shiraz Ahmad, Haseeb Valli, Nakulan Nanthakumar, Ibrahim T. Fazmin, Charlotte E. Edling, Christopher L.-H. Huang, Kamalan Jeevaratnam

**Affiliations:** a Faculty of Health and Medical Sciences, University of Surrey, GU2 7AL Guildford, United Kingdom; b School of Clinical Medicine, University of Cambridge, Cambridge, United Kingdom; c Physiological Laboratory and Department of Biochemistry, University of Cambridge, Cambridge, United Kingdom; d Bristol Medical School. University of Bristol, Bristol, United Kingdom

**Keywords:** Peroxisome proliferator activated receptor-γ (PPARγ), Coactivator-1 transcriptional coactivator (Pgc-1), Quantitative PCR, Ion channels, Mitochondria, Arrhythmias, Conduction velocity

## Abstract

Mitochondrial dysfunction underlying metabolic disorders such as obesity and diabetes mellitus is strongly associated with cardiac arrhythmias. Murine Pgc-1α^−/−^ hearts replicate disrupted mitochondrial function and model the associated pro-arrhythmic electrophysiological abnormalities. Quantitative PCR, western blotting and histological analysis were used to investigate the molecular basis of the electrophysiological changes associated with mitochondrial dysfunction. qPCR analysis implicated downregulation of genes related to Na^+^-K^+^ ATPase activity (Atp1b1), surface Ca^2+^ entry (Cacna1c), action potential repolarisation (Kcnn1), autonomic function (Adra1d, Adcy4, Pde4d, Prkar2a), and morphological properties (Myh6, Tbx3) in murine Pgc-1α^−/−^ ventricles. Western blotting revealed reduced Na_V_1.5 but normal Cx43 expression. Histological analysis revealed increased tissue fibrosis in the Pgc-1α^−/−^ ventricles. These present findings identify altered transcription amongst a strategically selected set of genes established as encoding proteins involved in cardiac electrophysiological activation and therefore potentially involved in alterations in ventricular activation and Ca^2+^ homeostasis in arrhythmic substrate associated with Pgc-1α deficiency. They complement and complete previous studies examining such expression characteristics in the atria and ventricles of Pgc-1 deficient murine hearts.

## 1 Introduction

Peroxisome proliferator activated receptor-γ (PPARγ) coactivator-1 transcriptional coactivators (Pgc-1), for which Pgc-1α and Pgc-1β constitute major subtypes, form an important family of regulators of mitochondrial biogenesis, mass and function, and expression of genes related to fatty acid β-oxidation, the tricarboxylic acid cycle and electron transport [1,2]. They are abundant in oxidative tissues including skeletal and cardiac muscle [3]. Pgc-1α is the most extensively investigated in relationship to control of such energy state and contractile function [2]. Its expression is strongly upregulated by physiological energy demands including exercise and fasting, as opposed to the background expression of other subtypes including Pgc-1β [1]. Pgc-1 expression is impaired in metabolic syndrome, obesity, insulin resistance and type 2 diabetes [4]. Cardiac tissue from both obese mice fed high fat diets and diabetic patients showed complex 1 abnormalities in their mitochondrial electron transport chains [5,6]. Obese and diabetic mice also showed reduced oxygen consumption rates, ATP generation and mitochondrial complexes I, III, and V protein expression [[7], [8], [9]]. Experimental evidence associates the energetic abnormalities accompanying these conditions with compromised cardiomyocyte glucose uptake, altered fatty acid metabolism, and impaired cardiac contractility [7,10].

Mitochondrial dysfunction is in turn implicated in pro-arrhythmic change. In combination with age [11,12], metabolic energetic disorders, themselves age-dependent, including physical inactivity [13], obesity [7,10,14], diabetes mellitus [15,16] and metabolic syndrome [17], conditions are associated only with clinical risks of ventricular arrhythmias and sudden cardiac death (SCD). SCD causes 4–5 million deaths per year worldwide [18] accounting for >5% of overall mortality [19], representing a major public health concern. The conditions also account for ~60% of current upward trends in the incidence of atrial fibrillation (AF) [20].

Murine systems have proven useful experimental models for studying both genotypic and phenotypic changes following genetic modifications associated with arrhythmic function [21], whether related to action potential activation and propagation, chronotropic responses to adrenergic stimulation, altered Ca^2+^ homeostasis or tissue fibrotic change [[22], [23], [24], [25], [26]]. Such systems have been used in a developing sequence of studies on Pgc-1α and Pgc-1β, atria and ventricles. The latter included molecular qPCR investigations of gene transcription, western blotting for protein expression, and histological analysis for fibrotic change. The present study complete this sequence of three previous reports bearing on molecular qPCR investigations of gene transcription in Pgc-1β atria and ventricles and Pgc-1α atria [[27], [28], [29]], investigating genes related to electrophysiological function in Pgc-1α ventricles.

## 2 Results

A selection of molecular techniques was used to investigate effects of the Pgc-1α^−/−^ genotypic model for mitochondrial dysfunction on expression of molecular determinants of cardiac arrhythmogenicity.

### 2.1 qPCR results

**Fig. 1:**
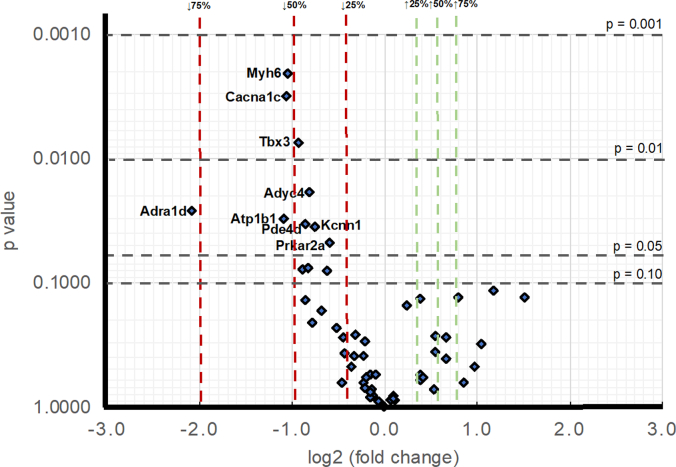
Stratifications of individual genes by fold changes and significance level in the comparison of Pgc-1α^−/−^ and WT ventricles. Volcano plot summarizing the effects of Pgc-1α−/− genotype on ventricular gene transcription. Magnitude of change in transcription levels as expressed as log2 transformed fold changes plotted against the level of statistical significance as the unadjusted *P*-values from independent Student's *t-*tests.

The genes studied were grouped by physiological function within the component processes underlying cardiac tissue excitability. Quantitative PCR utilising ThermoFisher custom Taqman pre-probed with the genes of interest compared transcriptional profiles of WT and Pgc-1α^−/−^ ventricles*.* Tables 1–10 summarises the qPCR results that gave mean genetic transcriptional -fold changes (means ±SEM) normalized to WT calculated using the *ΔΔC*_*t*_ method [30]. They summarize statistical assessments for transcriptional differences in both individual genes and functional groups of genes between WT and Pgc-1α^−/−^ ventricles, including controls confirming an absence of Pgc-1α mRNA expression (Table 10). Fig. 1 provides a volcano plot summarizing particularly noticeable transcriptional changes by virtue of effect sizes and statistical probabilities. It draws particular attention to downregulation of particularly genes related to Na^+^-K^+^ ATPase activity (Atp1b1), surface Ca^2+^ entry (Cacna1c), action potential repolarisation (Kcnn1), autonomic function (Adra1d, Adcy4, Pde4d, Prkar2a), and morphological properties (Myh6, Tbx3) in murine Pgc-1α^−/−^ ventricles.

#### 2.1.1 Genes encoding proteins underlying Na^+^-K^+^ ATPase activity

Energetically-dependent Na^+^-K^+^ ATPase-mediated active transport, in combination with the presence of intracellular impermeant ions, provide the transmembrane ionic gradients that contribute to maintenance of the resting potential [31,32]. We tested transcriptional activity of the α1 (*Atp1a1*) and α2 (*Atp1a2*) catalytic subunits and β1 accessory subunit (*Atp1b1*) of Na^+^-K^+^ ATPase. Table 1 demonstrates that Pgc-1α^−/−^ ventricles showed overall gene transcription levels within the gene group (*P* = 0.126), and *Atp1a1* (*P* = 0.804) and *Atp1a2* (*P* = 0.166) transcription levels indistinguishable from WT. However, they showed decreased *Atp1b1* transcription levels (*P* = 0.0301).

**Table 1:** Fold changes of RNA expression of genes underlying Na^+^-K^+^ ATPase activity.

Gene	Mean WT fold change (*n* = 3)	WT +/− SEM	Mean Pgc-1α^−/−^ fold change (n = 3)	Pgc-1α^−/−^ +/− SEM	*P* value (individual genes)	Group mean Pgc-1α^−/−^ fold change	Pgc-1α^−/−^ +/− SEM	P value (gene group)
*Atp1a1*	1	0.117	0.913	0.306	0.804	0.67	0.13	0.126
*Atp1a2*	1	0.184	0.623	0.124	0.166			
*Atp1b1*	1	0.156	0.468	0.0434	0.030			

#### 2.1.2 Genes encoding ion channels related to the resting membrane potential

The normal membrane resting potential is maintained primarily by outward K^+^ currents across inward rectifier K^+^ channels. We tested transcriptional activity of their underlying proteins including ATP-sensitive inward rectifier K^+^ channel Kir2.2 (*Kcnj12*), the ATP-binding cassette (ABC) transporter subunits members 8 (*Abcc8*) and 9 (*Abcc9*), the inwardly rectifying pore-forming K^+^ channels Kir6.1 (*Kcnj8*), Kir6.2 (*Kcnj11*) and Kir3.1 (*Kcnj3*), the G protein-activated inward rectifier potassium channel 4, Kir3.4 (*Kcnj5*), and the voltage gated Cl^−^ channel 3 (*Clcn3*). Table 2 demonstrates that Pgc-1α^−/−^ ventricles showed similar levels of overall gene transcription within the gene group as WT (*P* = 0.608). Pgc-1α^−/−^ ventricles also showed similar transcription levels through the individual genes, *Kcnj12* (*P* = 0.850), *Abcc8* (*P* = 0.116), *Abcc9* (*P* = 0.811), *Kcnj8* (*P* = 0.259), *Kcn11* (*P* = 0.634), *Kcnj3* (*P* = 0.0761), *Kcnj5* (*P* = 0.153), and *Clcn3* (*P* = 0.388) as WT.

**Table 2:** Fold changes of RNA expression of genes underlying ion channels relating to the resting membrane potential.

Gene	Mean WT fold change (*n* = 3)	WT +/− SEM	Mean Pgc-1α^−/−^ fold change (n = 3)	Pgc-1α^−/−^ +/− SEM	*P* value (individual genes)	Group mean Pgc-1α^−/−^ fold change	Pgc-1α^−/−^ +/− SEM	P value (gene group)
*Abcc8*	1	0.011	2.263	0.632	0.116	1.11	0.21	0.608
*Abcc9*	1	0.179	1.065	0.180	0.811			
*Kcnj11*	1	0.171	0.851	0.233	0.638			
*Kcnj12*	1	0.190	1.060	0.226	0.850			
*Kcnj3*	1	0.123	0.562	0.138	0.076			
*Kcnj5*	1	0.082	1.182	0.062	0.153			
*Kcnj8*	1	0.120	0.802	0.092	0.259			
*Clcn3*	1	0.138	0.855	0.059	0.388			

#### 2.1.3 Genes encoding ion channels relating to automaticity and action potential initiation and propagation

Pacemaker tissue expresses an inward depolarising pacemaker current (I_h_) carried by hyperpolarisation activated cyclic nucleotide gated (HCN) channels. HCN4, the predominantly expressed isoform, accounts for up to 90% of HCN generated current [33,34]. HCN4 knockouts show reduced I_f_, sinus pauses and bradycardia, with embryologic lethality in global HCN4 KO [33,35,36]. HCN1 KO shows reduced I_f_, to a lesser extent than HCN4 KO [37], with bradycardia, sinus dysrhythmia, and recurrent sinus pauses [37]. With the less studied HCN3 global HCN3 KO reduced I_f_ density by 30% and shortened action potential duration [38]. In contrast, roles of HCN2 remain controversial with low or absent HCN2 transcription and expression [34,39,40] and less marked phenotypes [41].

We tested transcriptional activity for HCN1 (*Hcn1*), HCN3 (*Hcn3*), and HCN4 (*Hcn4*) channels. Table 3 shows that Pgc-1α^−/−^ and WT ventricles showed similar levels of overall gene expression within the gene group (*P* = 0.191). They also showed similar expression levels of individual *Hcn1* (*P* = 0.833), *Hcn3* (*P* = 0.754), and *Hcn4* (*P* = 0.207) genes.

**Table 3:** Fold changes of RNA expression of genes underlying ion channels relating to the initiation of excitability.

Gene	Mean WT fold change (*n* = 3)	WT +/− SEM	Mean Pgc-1α^−/−^ fold change (n = 3)	Pgc-1α^−/−^ +/− SEM	*P* value (individual genes)	Group mean Pgc-1α^−/−^ fold change	Pgc-1α^−/−^ +/− SEM	P value (gene group)
*Hcn1*	1	0.370	0.897	0.269	0.833	0.79	0.11	0.191
*Hcn3*	1	0.217	0.900	0.204	0.754			
*Hcn4*	1	0.269	0.578	0.081	0.207			
*Scn5a*	1	0.190	1.060	0.226	0.850	0.77	0.22	0.476
*Scn7a*	1	0.123	0.562	0.138	0.076			

HCN-mediated depolarisation at pacemaker tissues then triggers Na^+^ channel dependent depolarisation responsible for the phase 0 upstroke of the cardiac action potential which spreads throughout the myocardium. We tested transcription activity of the α subunits of the voltage dependent Na_V_1.5 (*Scn5a*) and Na_V_2.1 (*Scn7a*). Table 3 demonstrates that Pgc-1α^−/−^ and WT ventricles showed similar levels of overall transcription within the gene group (*P* = 0.476), and similar expression levels of the individual *Scn5a* (*P* = 0.850) and *Scn7a* (*P* = 0.0761) genes.

#### 2.1.4 Genes underlying surface membrane Ca^2+^ channel activity

Membrane depolarisation opens surface membrane voltage gated Ca^2+^ channels responsible for the ventricular action potential plateau phase. These are involved in excitation-contraction coupling and can participate in pro-arrhythmic triggered, particularly early after depolarisation, events [42]. We tested transcriptional activity of the surface membrane L-type Ca^2+^ ion channels Ca_V_1.2 α1C subunit (*Cacna1c*) and Ca_V_1.3 α1D subunit (*Cacna1d*), the T-type channel Ca_V_3.1 α1G subunit (*Cacna1g*) and Ca_V_3.2 α1H (*Cacna1h*) subunits, and the accessory regulatory subunits β2 (*Cacnb2*), α2/δ1 (*Cacna2d1*) and α2/δ2 (*Cacna2d2*). Table 4 demonstrates that Pgc-1α^−/−^ and WT ventricles showed similar overall levels of gene transcription (*P* = 0.621), and of the individual *Cacna1d* (*P* = 0.129), *Cacna1g* (*P* = 0.605), *Cacna1h* (*P* = 0.977), *Cacnb2* (*P* = 0.892), *Cacna2d1* (*P* = 0.385), *Cacna2d2* (*P* = 0.640) genes. However, Pgc-1α^−/−^ showed lower *Cacna1c* expression (*P* = 0.00315).

**Table 4:** Fold changes of RNA expression of genes underlying surface Ca^2+^ homeostasis.

Gene	Mean WT fold change (*n* = 3)	WT +/− SEM	Mean Pgc-1α^−/−^ fold change (n = 3)	Pgc-11α^−/−^ +/− SEM	P value (individual genes)	Group mean Pgc-1α^−/−^ fold change	Pgc-1α^−/−^ +/− SEM	P value (gene group)
*Cacna1c*	1	0.016	0.477	0.081	0.003	1.16	0.30	0.621
*Cacna1d*	1	0.341	2.851	0.910	0.129			
*Cacna1g*	1	0.197	1.299	0.496	0.605			
*Cacna1h*	1	0.241	0.988	0.285	0.977			
*Cacna2d1*	1	0.199	0.790	0.084	0.385			
*Cacna2d2*	1	0.401	0.726	0.366	0.640			
*Cacnb2*	1	0.128	0.957	0.270	0.892			

#### 2.1.5 Genes underlying intracellular Ca^2+^ homeostasis

Excitation-contraction coupling involves alterations in cytosolic Ca^2+^ levels produced by Ca^2+^ release from and re-uptake into sarcoplasmic reticular Ca^2+^ stores. Abnormalities in these processes can result in arrhythmic triggering and substrate [43]. We tested transcriptional activity of the RyR isoforms RyR2 (*Ryr2*) and RyR3 (*Ryr3*), one of the cardiac SERCA isoforms (*Atp2a2*), the principal cardiac NCX (*Slc8a1*), and calsequestrin (*Casq2*). Table 5 demonstrates that Pgc-1α^−/−^ and WT ventricles showed similar levels of overall expression within the gene group (*P* = 0.564) and of the individual *Ryr2* (*P* = 0.0778), *Ryr3* (*P* = 0.472), *Atp2a2* (*P* = 0.355), *Slc8a1* (*P* = 0.553), and *Casq2* (*P* = 0.544) genes.

**Table 5:** Fold changes of RNA expression of genes underlying intracellular Ca^2+^ homeostasis.

Gene	Mean WT fold change (*n* = 3)	WT +/− SEM	Mean Pgc-1α^−/−^ fold change (n = 3)	Pgc-1α^−/−^ +/− SEM	P value (individual genes)	Group mean Pgc-1α^−/−^ fold change	Pgc-1α^−/−^ +/− SEM	P value (gene group)
*Atp2a2*	1	0.060	1.451	0.427	0.355	1.16	0.25	0.564
*Casq2*	1	0.021	0.895	0.157	0.544			
*Ryr2*	1	0.186	0.541	0.057	0.078			
*Ryr3*	1	0.208	1.960	1.194	0.472			
*Slc8a1*	1	0.078	0.932	0.072	0.553			

#### 2.1.6 Genes underlying ion channels mediating action potential repolarisation

Outward membrane current resulting from K^+^ channel opening drives action potential repolarisation, controlling action potential duration whose prolongation is associated with arrhythmic tendency. We tested transcriptional activity of the voltage-sensitive transient outward current I_to_ carried by K_V_1.4 subfamily D member 4 (*Kcna4*) and K_V_4.3 subfamily D member 3 (*Kcnd3*), voltage-gated K^+^ channel, mediating the rapid K^+^ current I_Kr_, K_V_11.1 subfamily H member 2 (*Kcnh2*), the Ca^2+^-activated K^+^ channels K_Ca_2.1, subfamily N member 1 (*Kcnn1*), and K_Ca_2.2, subfamily N member 2 (*Kcnn2*), acid-sensitive K^+^ channel subfamily K member 3 (*Kcnk3*), and the regulatory KCNE1 subunit (*Kcne11*). Table 6 demonstrates that Pgc-1α^−/−^ and WT ventricles showed similar overall transcription levels within the gene group (*P* = 0.920) and similar transcription of the individual *Kcna4* (*P* = 0.310), *Kcnd3* (*P* = 0.0789), *Kcnh2* (*P* = 0.275), *Kcnn2* (*P* = 0.881), *Kcnk3* (*P* = 0.872), *Kcne11* (P = 0.275) genes. However, Pgc-1α^−/−^ ventricles showed lower *Kcnn1* transcription levels than WT (P = 0. 0350).

**Table 6:** Fold changes of RNA expression of genes underlying ion channels relating to repolarisation.

Gene	Mean WT fold change (*n* = 3)	WT +/− SEM	Mean Pgc-1α^−/−^ fold change (n = 3)	Pgc-1α^−/−^ +/− SEM	*P* value (individual genes)	Group mean Pgc-1α^−/−^ fold change	Pgc-1α^−/−^ +/− SEM	P value (gene group)
*Kcna4*	1	0.281	2.051	0.860	0.310	1.02	0.22	0.920
*Kcnd3*	1	0.112	0.646	0.101	0.079			
*Kcnh2*	1	0.155	0.731	0.147	0.275			
*Kcnk3*	1	0.151	1.041	0.187	0.872			
*Kcnn1*	1	0.110	0.592	0.070	0.0350			
*Kcnn2*	1	0.156	1.080	0.473	0.881			
*Kcne1l*	1	0.175	1.575	0.420	0.275			

#### 2.1.7 Genes encoding adrenergic and cholinergic receptors

Cardiac electrophysiology, and hence arrhythmic tendency, is heavily modulated by sympathetic and parasympathetic activity, and targeting autonomic receptors is a mainstay of clinical anti-arrhythmic management. We tested transcriptional activity in the main cardiac muscarinic acetylcholine receptor M2 (*Chrm2*), the α1-adrenoreceptor subtypes α1A (*Adra1a*), α1B (*Adra1b*), and α1D (*Adra1d*), and the β1 (*Adrb1*) and β2 (*Adrb2*) adrenergic receptor subtypes. Table 7 shows that Pgc-1α^−/−^ and WT ventricles showed similar overall levels of gene transcription (*P* = 0.920) and of transcription of individual *Adra1a* (*P* = 0.986), *Adra1b* (*P* = 0.575), *Adrb1* (*P* = 0.702), *Adrb2* (*P* = 0.543), and *Chrm2* (*P* = 0.992) genes. However, Pgc-1α^−/−^ ventricles showed decreased *Adra1d* expression compared to WT (P = 0. 0261) potentially disrupting autonomic modulation.

**Table 7:** Fold changes of RNA expression of genes underlying adrenergic and cholinergic receptors.

Gene	Mean WT fold change (*n* = 3)	WT +/− SEM	Mean Pgc-1α^−/−^ fold change (n = 3)	Pgc-1α^−/−^ +/− SEM	*P* value (individual genes)	Group mean Pgc-1α^−/−^ fold change	Pgc-1α^−/−^ +/− SEM	P value (gene group)
*Adra1a*	1	0.114	0.9950	0.248	0.986	0.88	0.14	0.432
*Adra1b*	1	0.114	0.873	0.175	0.575			
*Adra1d*	1	0.206	0.236	0.082	0.0261			
*Adrb1*	1	0.198	0.862	0.271	0.702			
*Adrb2*	1	0.226	1.302	0.395	0.543			
*Chrm2*	1	0.137	0.996	0.388	0.992			

#### 2.1.8 Genes underlying the cAMP pathway

Adrenergic and cholinergic receptors are G-protein coupled receptors (GPCRs) which utilise the intracellular cAMP cascade to transduce the signal into downstream effects on cardiomyocyte contractility and electrophysiological function. We tested transcriptional activity of adenylyl cyclase types 4 (*Adcy4*) and 5 (*Adcy5*), the cGMP-dependent and cAMP-specific 3′,5′-cyclic phosphodiesterases 2A (*Pde2a*) and 4D (*Pde4d*), the protein kinase A (PKA) catalytic α-subunit (*Prkaca*), the cAMP-dependent protein kinase regulatory subunits type I-α (*Prkar1a*), II-α (*Prkar2a*) and II-β (*Prkar2b*), and the Ca^2+^/calmodulin-dependent protein kinase, type II-δ (*Camk2d*). Table 8 demonstrates that Pgc-1α^−/−^ and WT ventricles showed similar overall levels of gene transcription (*P* = 0.941) and similar transcription levels of the individual *Adcy5* (*P* = 0.269), *Camk2d* (*P* = 0.363), *Pde2a* (*P* = 0.232), *Prkaca* (*P* = 0.723), *Prkar1a* (*P* = 0.132), and *Prkar2b* (*P* = 0.406) genes. Pgc-1α^−/−^ ventricles showed decreased *Adcy4* (*P* = 0.0186), *Pde4d* (*P* = 0.0338), and *Prkar2a* (*P* = 0.0474 transcription levels compared to WT compatible with possible disruptions in autonomic function.

**Table 8:** Fold changes of RNA expression of genes underlying the cAMP pathway.

Gene	Mean WT fold change (*n* = 3)	WT +/− SEM	Mean Pgc-1α^−/−^ fold change (n = 3)	Pgc-1α^−/−^ +/− SEM	P value (individual genes)	Group mean Pgc-1α^−/−^ fold change	Pgc-1α^−/−^ +/− SEM	P value (gene group)
*Adcy4*	1	0.091	0.568	0.067	0.019	0.99	0.16	0.941
*Adcy5*	1	0.122	1.461	0.338	0.269			
*Camk2d*	1	0.229	0.742	0.105	0.364			
*Pde2a*	1	0.188	0.695	0.108	0.232			
*Pde4d*	1	0.090	0.551	0.110	0.034			
*Prkaca*	1	0.162	0.904	0.192	0.723			
*Prkar1a*	1	0.167	1.731	0.349	0.132			
*Prkar2a*	1	0.071	0.661	0.097	0.047			
*Prkar2b*	1	0.332	1.580	0.529	0.406			

#### 2.1.9 Genes underlying fibrotic markers

Cardiac fibrosis is a major determinant of conduction velocity and arrhythmic tendency and has been associated with metabolic disorders. We tested transcriptional activity of fibrotic marker cytokine transforming growth factor β1 isoform TGF-β1 (*Tgfb1*), the major component of type I collagen (*Col1a1*), the collagen type III α1 chain (*Col3a1*), and the gap junction forming proteins δ3 Connexin 30.2 (*Gjd3*). Table 9 demonstrates that Pgc-1α^−/−^ and WT showed similar levels of overall gene transcription within the gene group (*P* = 0.478) and similar transcription levels of the individual *Tgfb1* (*P* = 0.297), *Col1a1* (*P* = 0.471), *Col3a1* (*P* = 0.569), and *Gjd3* (*P* = 0.630) genes.

**Table 9:** Fold changes of RNA expression of genes underlying fibrotic markers.

Gene	Mean WT fold change (*n* = 3)	WT +/− SEM	Mean Pgc-1α^−/−^ fold change (n = 3)	Pgc-1α^−/−^ +/− SEM	*P* value (individual genes)	Group mean Pgc-1α^−/−^ fold change	Pgc-1α^−/−^ +/− SEM	P value (gene group)
*Col1a1*	1	0.178	0.774	0.221	0.471	1.19	0.24	0.478
*Col3a1*	1	0.370	1.327	0.374	0.569			
*Gjd3*	1	0.596	1.806	1.429	0.630			
*Tgfb1*	1	0.103	0.862	0.052	0.297			

#### 2.1.10 Miscellaneous genes encoding proteins with developmental, morphological or other properties

Further genes not directly related to tissue excitability but nonetheless associated with arrhythmic tendency include the transcriptional repressor Tbx3 (*Tbx3*), the non-specific ion channel TRPC1 (*Trpc1*), the myosin heavy chain α isoform (*Myh6*), and the natriuretic peptide A (*Nppa*). In addition, we surveyed expression of control, *Ppargc1a* and *Ppargc1b* genes encoding Pgc-1α and β, respectively. Table 10 demonstrates that Pgc-1α^−/−^ and WT ventricles showed similar overall transcription levels within the gene group (*P* = 0.448) and similar transcription levels of the individual *Nppa* (*P* = 0.130), *Trpc1* (*P* = 0.715), and *Ppargc1b* (*P* = 0.134) genes. Pgc-1α^−/−^ ventricles showed decreased *Tbx3* (*P* = 0.00735) and *Myh6* (*P* = 0.00207) transcription compared to the WT and an absence of *Ppargc1a* transcription.

**Table 10:** Fold changes of other miscellaneous genes.

Gene	Mean WT fold change (*n* = 3)	WT +/− SEM	Mean Pgc-1α^−/−^ fold change (n = 3)	Pgc-1α^−/−^ +/− SEM	*P* value (individual genes)	Group mean Pgc-1α^−/−^ fold change	Pgc-1α^−/−^ +/− SEM	P value (gene group)
*Myh6*	1	0.061	0.482	0.040	0.002	2.23	1.49	0.448
*Nppa*	1	0.204	9.621	4.535	0.130			
*Ppargc1a*	1	0.235	0	0	0			
*Ppargc1b*	1	0.057	1.305	0.153	0.135			
*Tbx3*	1	0.093	0.526	0.017	0.007			
*Trpc1*	1	0.538	1.451	1.016	0.715			

### 2.2 Western blot expression of protein determinants of action potential conduction velocity

**Fig. 2:**
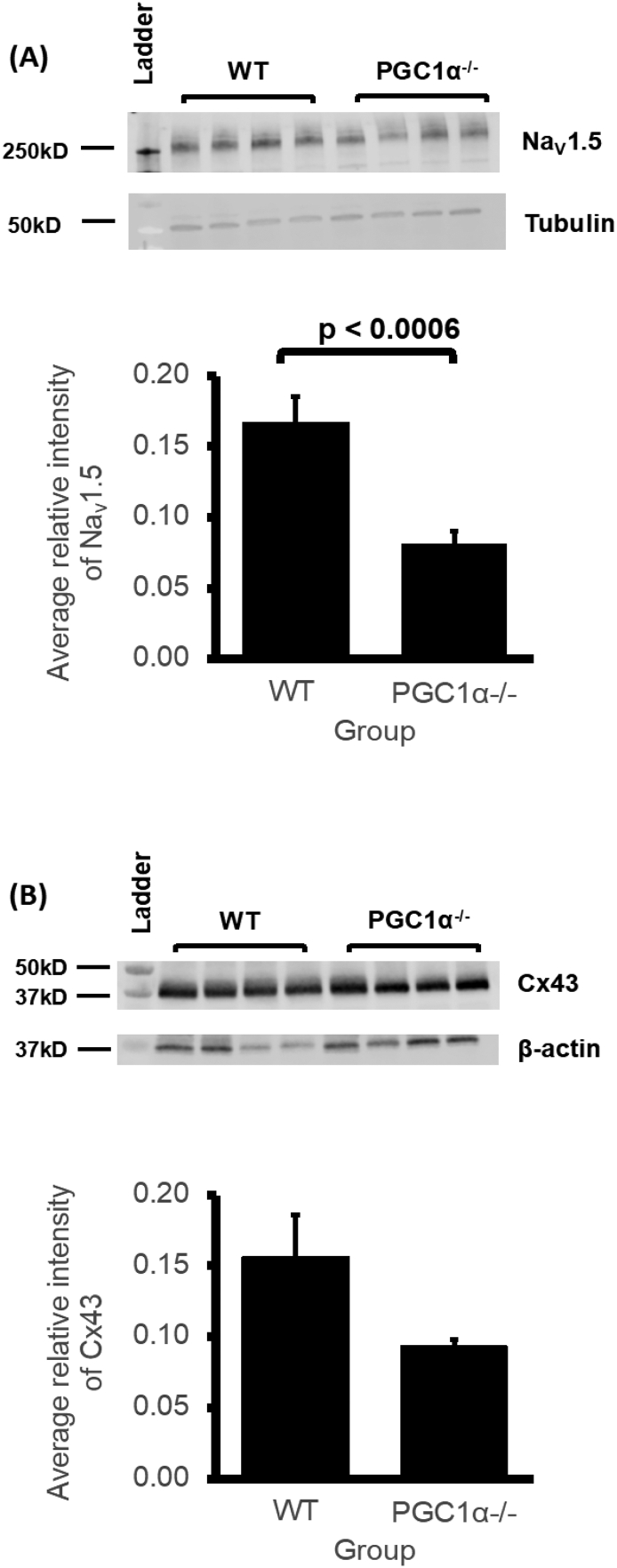
(A) Na_V_1.5 expression levels in WT (*N* = 4) and Pgc-1α^−/−^ (N = 4) ventricles obtained by densitometric analysis and their representative Western blots including the housekeeping protein. (B) Cx43 expression levels in WT (N = 4) and Pgc-1α^−/−^ (N = 4) ventricles obtained by densitometric analysis and their representative Western blots including the housekeeping protein. Initial bicinchoninic acid (BCA) assay estimate of lysate protein concentration determined lysate volumes ensuring equal amounts of protein in each well. Measurements made along with a further control housekeeping protein (β -tubulin (A), β-actin (B)) not affected by the difference in genotype, for normalization of the protein of interest to control for any residual variation in amount of protein loaded in each well.

Amongst the above gene expression patterns examined, previous studies had reported situations in which normal Nav1.5 mRNA expression on qPCR accompanied a reduced protein expression on western blotting, suggesting possible specific post-transcriptional regulatory effects. These had both involved atrial [27] and ventricular Pgc-1β^−/−^ Nav1.5 gene [29] as well as protein expression [44], as well as examples in other systems [45,46]. There were similar differences involving gja mRNA markers and Cx40 and Cx43 protein expression in the same reports. We therefore went on to investigate protein content levels of Na_V_1.5 and Cx43 proteins in Pgc-1α^−/−^ and WT ventricles, analysing Western blots of tissue lysates. Fig. 2A shows a significantly reduced Na_V_1.5 protein expression in Pgc-1α^−/−^ compared to WT ventricles (*P* = 0.0006), in contrast to the qPCR mRNA results. In contrast, Fig. 2B demonstrates that there were no significant differences in expression levels of Cx43 protein between Pgc-1α^−/−^ and WT ventricles (*P* = 0.057). Both Na_V_1.5 and Cx43 proteins are important determinants of action potential conduction velocity. Na_V_1.5 mediated fast inward Na^+^ current (I_Na_) determines the maximum rate of membrane depolarisation (d*V*/d*t*)_max_; gap junction connexin (Cx) proteins at the intercalated discs are determinants of axial resistance (r_a_) to local circuit current flow determining intercellular coupling [44,47], for which Cx43 is the dominant ventricular isoform [48].

### 2.3 Histological analysis of cardiac tissue fibrosis

**Fig. 3:**
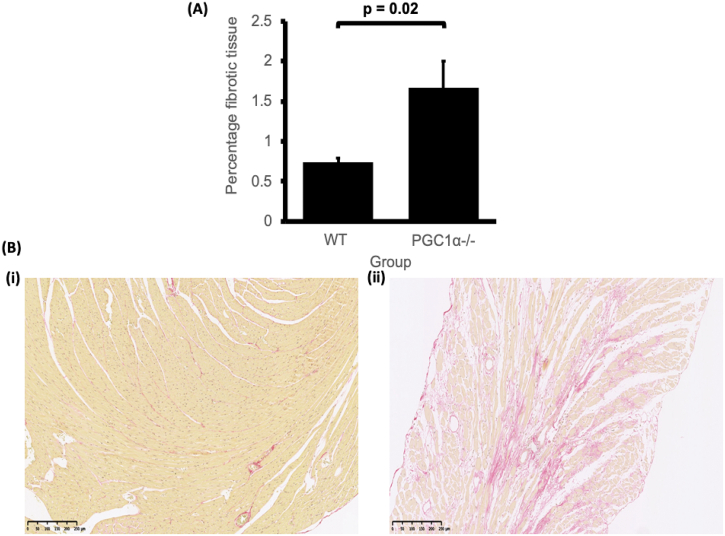
(A) Shows percentage fibrotic tissue in WT (*N* = 8) and Pgc-1α^−/−^ (*N* = 9) ventricles obtained by histological analysis. (B) Shows representative example of the absence of fibrotic change in old WT ventricles (i) and the presence of fibrotic change in old Pgc-1α^−/−^ ventricles obtained by histological analysis (length of scale bar: 250 μm).

Conduction velocity is also influenced by cardiac fibrosis through its effects on increasing cell effective capacitances, *c*_m_, through myocyte-fibroblast coupling and in increasing *r*_a_ through decreasing Cx expression by myocyte-myocyte decoupling [21]. Yet Section 2.1.9 reported qPCR results which revealed no change in transcription of molecular markers of fibrosis. However, the absence of increased transcription in cardiac fibrosis related genes, particularly TGF-β1, parallels previous reported histological incidences of increased tissue fibrosis without increased transcription of corresponding fibrosis-related genes [[27], [28], [29],^43^]. Thus, murine Pgc-1β deficient atria and ventricles similarly showed unaltered transcription of cardiac fibrosis genes including *Tgfb1* and *Col3a1* despite histological evidence of fibrotic change [23,26,27,29]. Other studies investigating the role of such genes in cardiac fibrosis had demonstrated that their expression increases only transiently and hence may not be detected by molecular assays beyond a particular point in time [49,50]. In parallel with the previous reports, we accordingly performed a histological analysis for such changes. in Pgc-1α^−/−^ and WT ventricles. Fig. 3 shows percentage fibrotic tissue in Pgc-1α^−/−^ and WT ventricles. Pgc-1α^−/−^ ventricles show significantly increased tissue fibrosis compared to WT ventricles (*P* = 0.0186).

## 3 Discussion

Metabolic disorders associated with mitochondrial dysfunction and energetic deficiency including obesity and diabetes mellitus, represent major public health concerns that include increased cardiac arrhythmic and SCD risks [[14], [15], [16]] through molecular mechanisms still poorly understood. We explore these using a Pgc-1α−/− murine model. PGC-1α is involved in expression of genes involved in oxidative phosphorylation important in energy metabolism. It is abundantly expressed in actively oxidative tissues often characterized by abundant mitochondria. The latter include brown adipose tissue, and cardiac and skeletal muscle. Its deficiency is associated with impaired mitochondrial maximal oxygen consumption and ATP synthesis. It appears important to cardiac adjustments that require increased ATP and work output in response to physiological stimuli.

First, quantitative PCR examined transcriptional profiles of groups of genes underlying aspects of electrophysiological function. These related to Na^+^-K^+^ ATPase activity, resting membrane potential, initiation of excitation, surface membrane Ca^2+^ permeation and intracellular Ca^2+^ homeostasis, membrane repolarisation, autonomic and cAMP dependent signalling, tissue fibrotic markers, and morphological remodelling changes. Second, Western blotting studies explored Na_V_1.5 and Cx43 protein expression and histological methods assessed myocardial fibrosis, factors directly bearing on cardiac conduction velocity changes implicated in re-entrant substrate [21,47]. Thus, maximum action potential upstroke rates (d*V*/d*t*)_max_ are determined by Nav1.5 mediated fast Na^+^ current (I_Na_) [21,47]. The resulting local circuit current flow between coupled cardiomyocytes is determined by gap junction connexin (Cx) channel expression which influences axial resistance (*r*_a_) [21,47]. The degree of cardiac fibrosis influences not only coupling between cardiomyocytes [51,52], but also their effective membrane capacitances (c_m_) altered through Cx-mediated myocyte-fibroblast coupling [53,54].

Of biomolecules related to the background ionic gradients supporting excitable activity, Pgc-1α^−/−^ ventricles demonstrated reduced regulatory *Atp1b1*, but normal catalytic *Atp1a1* or *Atp1a2*, subunit transcription required for Na^+^-K^+^ ATPase activity, compared to WT. This complements previous reports that Pgc-1α^−/−^ atria showed reduced transcription of all three catalytic and regulatory subunits [28].

Of biomolecules related to action potential initiation, Pgc-1α^−/−^ and WT ventricles showed similar transcription levels of HCN channels in contrast to previous reports of reduced atrial *HCN1* and *HCN4* channel transcription [28]. Pgc-1α^−/−^ ventricles also showed similar *Scn5a* gene but decreased Na_V_1.5 protein expression levels compared to WT. Previously, Pgc-1α^−/−^ atria had been shown to have reductions in both gene and protein levels of Na_V_1.5 [28]. However, Pgc-1α^−/−^ and WT ventricles showed similar Cx43 connexin protein expression [48], in agreement with previous findings in Pgc-1α^−/−^ atria and Pgc-1β^−/−^ ventricles [28,44]. Finally, histological analysis revealed greater tissue fibrosis in Pgc-1α^−/−^ than WT ventricles in parallel with previous reports on Pgc-1β^−/−^ ventricles [26], with possible implications for cardiomyocyte-myofibroblast coupling [55,56]. These changes could arise from increased oxidative stress and ROS production acting on gene or protein expression and promoting TGF-β activity [26,57,58].

Of genes related to surface Ca^2+^ channel activity, Pgc-1α^−/−^ ventricles showed reduced *Cacna1c* transcription encoding cardiac L-type Ca^2+^ channel Ca_V_1.2 compared to WT. Previous studies in Pgc-1α^−/−^ atria had similarly reported reduced transcription of *Cacna1c*, *Cacna2d2*, and *Cacna2d1* [28]. Pgc-1β^−/−^ myocytes had shown no alteration in such transcription levels [22]. Pgc-1α^−/−^ ventricles and WT showed similar expression levels of genes underlying sarcoplasmic reticular regulation of intracellular Ca^2+^ homeostasis. Previous findings had also suggested that Pgc-1α^−/−^ atria showed decreased *Casq2* transcription suggesting reduced SR Ca^2+^ storage capacity [28], and that Pgc-1β^−/−^ ventricles showed increased *CAMKII*, *RyR2*, *CASQ1* transcription [22]. Other mitochondrial dysfunction models have shown increased RyR2 and NCX, and reduced SERCA expression [59].

Pgc-1α^−/−^ ventricles showed similar transcription levels of K^+^ channels underlying repolarisation apart from a reduced *Kcnn1* transcription compared to WT. This parallels previous reports that Pgc-1α^−/−^ atria showed no change in transcription levels of such genes [28].

Of genes underlying adrenergic and cholinergic and their associated intracellular signalling, Pgc-1α^−/−^ ventricles showed reduced *Adra1b*, *Adcy4*, *Pde4* and *Prkar2a* transcription relative to WT. Pgc-1α^−/−^ atria had similarly shown decreased *Adra1b* and *Adcy4* gene transcription levels [28]. This may parallel the disrupted autonomic responses in Pgc-1α^−/−^ hearts. In addition, α1D adrenoreceptor (*Adra1b*; α1D AR) signalling through G_q/11_ pathway is thought to play an important in the protective and adaptive functions in the heart preventing pathological remodelling [60]. Its deficiency could potentially contribute to the fibrotic changes in Pgc-1α^−/−^ ventricles [60,61].

Finally, of a number of genes related to developmental and morphological changes, Pgc-1α^−/−^ ventricles showed reduced Tbx3 and Myh6 transcription. The transcriptional repressor Tbx3 is an important cardiac development regulator for differentiation of cardiac conduction systems [62], whose loss-of-function is associated with arrhythmic phenotypes [63]. *Myh6* encodes the myosin heavy chain (MHC) isoforms α-MHC crucial in the sarcomere organisation and muscle contraction [64], mutations in which are associated with sinus node disorder [64,65].”

**Fig. 4:**
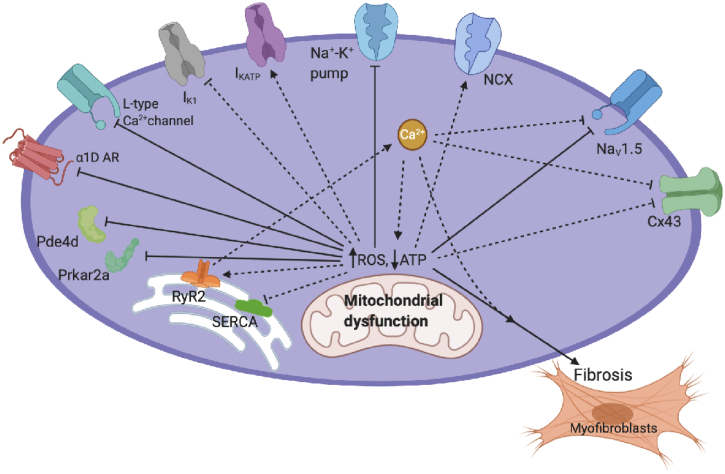
Molecules implicated in electrophysiological changes with mitochondrial dysfunction. Solid lines: changes suggested in the present experiments. Dotted lines: other relevant changes discussed in the paper reported in other experiments. Arrow-end: positive effects increasing activity/expression; straight horizontal-end: negative effects decreasing activity/ expression. Created using BioRender.com.

These present studies thus identified patterns of cardiac ventricular transcriptional changes amongst a set of genes strategically selected for proteins involved in electrophysiological activation and Ca^2+^ homeostasis and consequent arrhythmic tendency associated with Pgc-1α deficiency (Fig. 4). These complement previous studies examining corresponding expressional changes in the atria and ventricles of Pgc-1β deficient murine hearts. They will provide a background for future explorations for electrophysiological correlates for these gene alterations. The latter would include explorations of resting membrane potentials, chronotropic sinus properties [34], action potential activation [25,26] and conduction velocities [[24], [25], [26]]. The latter are potentially modifiable by altered cytosolic Ca^2+^ signals and the effects upon these of adrenergic challenge [22]. Biochemical studies could examine for lipodomic, energetic and oxidative, including reactive oxidative species (ROS), phenotypes. Studies in intact animals could examine ventricular recovery times and QT intervals following dobutamine stimulation [[24], [25], [26]] and exercise and dobutaminergic responses. Some of these could make comparisons between the left and right ventricles in view of some of their contrasting disease phenotypes.

## 4 Materials and methods

### 4.1 Animals

Experimental protocols were approved under the UK Home Office regulations [Animals (Scientific Procedures) Act 1986 Amendment Regulations 2012] following ethical review by the University of Cambridge Animal Welfare and Ethical Review Body (AWERB) and conducted under a designated project license. The experiments also conformed to the Guide for the Care and Use of Laboratory Animals, U.S. National Institutes of Health (NIH Publication No. 85-23, revised 1996). An animal house maintained at 21 °C was used for the mice, with 12-h light/dark cycles. The mice had sterile chow (RM3 Maintenance Diet, SDS, Witham, Essex, UK) and free access to water, bedding and environmental stimuli. Mice were sacrificed by cervical dislocation and no anaesthetic or surgical procedures were required. Wild-type (WT) C57/B6 and Pgc-1α^−/−^ (The Jackson Laboratory, Bar Harbor, ME, USA) adult mice were bred for the experimental protocols Mice were bred on a C57/B6 background to avoid possible strain-related confounds. The mice were divided into WT and Pgc-1α^−/−^, with ages defined in each experiment.

### 4.2 Quantitative PCR

WT (*N* = 3, mean age = 18 months) and Pgc-1α^−/−^ (N = 3, mean age = 16.7 months) RNA was extracted from fresh frozen tissues, stored in −80 °C, with the Qiagen RNeasy mini Plus kit (Qiagen, Manchester, UK) similar to previously reported protocols [27,28]. Cardiac ventricular tissue was weighed and placed on ice and 30 mg of tissue was subsequently homogenized in RLT buffer supplemented with β-mercaptoethanol with a Stuart handheld homogenizer until completely smooth. Genomic DNA was eliminated by centrifugation through a column supplied with the kit prior to extraction of the RNA according to the manufacturer's protocol. RNA integrity was assessed by using an Agilent bioanalyzer to obtain RNA integrity numbers (RIN) according to the manufacturer's protocol (Agilent Technologies, Santa Clara, CA, USA). RNA samples with RINs over 8 were used for the study. cDNA was prepared from RNA with High Capacity cDNA Reverse Transcription Kit (Applied Biosystems, Waltham, MA, USA) according to manufacturer's instructions. The efficiency of the protocol was tested by serial dilution technique with the samples run using a SYBRgreen qPCR confirming equal efficiency over a range of RNA concentrations. SYBRgreen qPCR also confirmed cDNA negative for genomic DNA contamination. In assays using Thermo Fisher custom Taqman array cards, each custom-made card contained 64 pre-validated assays in triplicate with a reaction volume of 1 μl The cards were run according to instructions specific for the cards. Briefly, the cDNA (100 ng/well) was mixed with 2× Mastermix from Thermo Fisher Scientific (Waltham, MA, USA), 100 μl was loaded in each well slot on the cards. The cards were then spun down and sealed and run on a Quant 7 cycler. The amplification conditions were: 50 °C for 2 min and 95 °C for 10 min for the initial DNA melting and inactivation of the RT reaction, followed by 40 cycles at 95 °C for 15 s and 60 °C for 60 s. Analysis of the Taqman array card data was performed by using the Quant studio software and Microsoft Excel (Microsoft Corporation, Redmond, WA, USA) by calculating fold changes with the delta-delta-CT method as described by Livak and Schmittgen [30]. The threshold was set at 0.2 fluorescence units and the baseline range was set to automatic assignment. The geometric mean of the Cq values for the genes HPRT, Gapdh and ActinB were used as references and amplifications were calculated with the regression threshold and baseline subtraction curve fit auto settings with the BioRad CFX manager software. The statistical analysis used paired Student's *t*-tests to assess for differences in expression within functional gene groups and unpaired Student's *t*-test to compare for differences in expression of individual genes between WT and Pgc-1α^−/−^ ventricles. Statistical significance was set at *P* < 0.05. Prior to the present and all the following statistical tests, the Shapiro-Wilk test [66] was performed on all of the WT and Pgc-1α^−/−^ data obtained by western blotting (*N* = 4) and qPCR (*N* = 3) and no significant results were found (*P* > 0.05) in any of the tests. This justified selection of parametric tests, which have more statistical power than their non-parametric equivalents, whilst recognizing that an important limitation of small sample sizes is reduced statistical power and increased type II error rates [67].

### 4.3 Western blotting

The present experiments followed similar previously reported protocols [28,44]. WT (N = 4, mean age = 18.8 months) and Pgc-1α^−/−^ (N = 4, mean age = 21.5 months) ventricles were homogenized using a Stuart® tissue homogenizer (Cole-Parmer, UK) in 900 μl of lysis buffer (150 mM NaCl, 25 mM tris(hydroxymethyl)aminomethane (tris), pH 7–8, 1% Triton-X100 detergent, 5 mM ethylenediaminetetraacetic acid (EDTA) and Roche® cOmplete™ mini protease inhibitor (Merck KGaA, Darmstadt, Germany)). After a 20-min centrifugation at 12,000 RPM, the clear lysate was assayed for protein concentration using a bicinchoninic acid (BCA) assay (Thermo Scientific Microplate BCA Protein Assay Kit #23252: manufacturer recommended protocol).

For sodium dodecyl sulphate polyacrylamide gel electrophoresis (SDS-PAGE) the samples were incubated with a loading buffer (12.8 ml tris, pH 6.8, 3.2 g sodium dodecyl sulphate (SDS), 1.85 g dithiothreitol (DTT), 16 ml 100% glycerol, bromophenol blue, 11.2 ml H_2_O) in the ratio of 3:1 volume of clear lysate to loading buffer. The mixtures were heated at 70 °C for 5 min and then loaded into Mini-Protean TGX™ (Bio-Rad, Watford, UK), 4–15% acrylamide gradient, precast gel wells (20 μg for Nav1.5 blots and 30 μg for Cx43 blots) with coloured protein ladder (Precision Plus Protein™ Dual Colour Standards, Bio-Rad, UK) used to estimate molecular weights of protein bands and the gel exposed to a potential of 120 V for 30 min, then 250 V for 20 min. Proteins were electrophoretically transferred from the gel onto polyvinylidene fluoride (PVDF) membranes (Immobilon™ PVDF membrane, Merck KGaA, Germany) using Trans-Blot® Turbo™ (Bio-Rad, UK) at settings of 1.3 A current and 25 V potential for 10 min. Odyssey® blocking buffer (Li-Cor Biosciences, Cambridge, UK) was used to block the PVDF membranes for one hour at room temperature. Membranes were then rinsed with PBS-T (0.1% Tween) and incubated with primary antibody diluted in Odyssey® blocking buffer diluted 33% in PBS-T overnight at 4 °C. The primary antibodies used were Na_V_1.5 (Cell Signalling Technology, London, UK), Cx43 (Sigma-Aldrich Company Ltd., Gillingham, UK) and β-tubulin (Abcam, Cambridge, UK). The membranes were washed three times and then incubated with secondary antibodies diluted in Odyssey® blocking buffer diluted 33% in PBS-T at room temperature for 45 min. The membranes were then exposed to secondary antibodies conjugated with dyes for near infrared fluorescence (NIF). Imaging of the blots utilised the Odyssey® Fc imaging system (Li-Cor Biosciences, Cambridge, UK), which measured emission from the secondary antibodies at 600 and 800 nm. Image Studio™ software (Image Studio 4.0, Li-Cor Biosciences, Cambridge, UK) was used to quantify the protein band intensity and subtract the background signal, and then express this relative to the control, β-tubulin, signal. Statistical analysis utilised the unpaired Student's *t*-test. Statistical significance was set at *P* < 0.05.

### 4.4 Histological analysis

Measurement of cardiac fibrosis followed methods used in previous studies [68]. WT (*N* = 8, mean age = 17.4 months) and Pgc-1α^−/−^ (*N* = 9, mean age = 18.7 months) ventricles were used for histological analysis. Custom Krebs buffer (containing, in mM, NaCl 119, NaHCO_3_ 25, KCl 4, KH_2_PO_4_ 1.2, MgCl_2_ 1, CaCl_2_ 1.8, glucose 10 and Na-pyruvate 2, pH 7.4, 95% O_2_/5% CO_2_; British Oxygen Company, Manchester, UK) was used to flush the isolated hearts. Hearts were then perfused with 4% buffered formalin for 5 min and then kept in formalin overnight. Following fixation, gross transverse 7 μm thick sections were taken. This was followed by routine tissue processing and paraffin embedding. The sections were then stained using a Sirius red protocol, involving a series of immersions: xylene for 2 min, new batch of xylene for 2 min, 95%, 70% and 50% ethanol for 2 min each, Weigerts Haemotoxylin for 8 min, running water for 10 min, picro-sirius red solution for 1 h, acidified water for 16 dips, 3 changes of 100% ethanol for 1-min each and xylene for 3 dips. The slides were then mounted and subsequently viewed, magnified, and digitally acquired using the Nano Zoomer 2.0 Digital Pathology system (Hamamatsu, Hertfordshire, UK). Following magnification, a custom-made 17 cm × 30 cm morphometric grid, consisting of square boxes of dimension 1 cm × 1 cm, corresponding to an approximate 0.2 mm × 0.2 mm area of tissue, was then superimposed on each photomicrograph. If a square occupied either completely or partially by cardiac tissue showed the presence of fibrosis, it was counted and then the number of these squares was then expressed as a percentage of total cardiac tissue area for each heart. Statistical analysis utilised the unpaired Student's *t*-test. Statistical significance was set at *P* < 0.05.

## Author contributions

Conceptualization, TF, CE, CLHH and KJ; methodology, KS, SA, HV, KC; validation KS, SA, HV, KC; formal analysis, KS, KC; data curation, KS, KC, AP, NNK, TF and CEE, writing—original draft preparation, KS and KC; writing—review and editing, KS, KC, AP, NNK, CEE, CLHH and KJ; supervision, TF, CEE, CLHH and KJ; project administration, CEE and KJ; funding acquisition, CLHH and KJ. All authors have read and agreed to the published version of the manuscript.

## Funding

This work was supported by the 10.13039/501100000265Medical Research Council [grant number MR/M001288/1]; the 10.13039/100010269Wellcome Trust [grant number 105727/Z/14/Z]; the 10.13039/501100000274British Heart Foundation [grant numbers PG/14/79/31102, PG/14/79/31102].

## Declaration of Competing Interest

The authors declare no conflict of interest. The funders had no role in the design of the study; in the collection, analyses, or interpretation of data; in the writing of the manuscript, or in the decision to publish the results.
